# G Protein-Coupled Receptors Regulated by Membrane Potential

**DOI:** 10.3390/ijms232213988

**Published:** 2022-11-12

**Authors:** Dekel David, Ziv Bentulila, Merav Tauber, Yair Ben-Chaim

**Affiliations:** Department of Natural and Life Sciences, The Open University of Israel, Ra’anana 4353701, Israel

**Keywords:** G-protein coupled receptors, voltage dependence, membrane potential, voltage sensor

## Abstract

G protein-coupled receptors (GPCRs) are involved in a vast majority of signal transduction processes. Although they span the cell membrane, they have not been considered to be regulated by the membrane potential. Numerous studies over the last two decades have demonstrated that several GPCRs, including muscarinic, adrenergic, dopaminergic, and glutamatergic receptors, are voltage regulated. Following these observations, an effort was made to elucidate the molecular basis for this regulatory effect. In this review, we will describe the advances in understanding the voltage dependence of GPCRs, the suggested molecular mechanisms that underlie this phenomenon, and the possible physiological roles that it may play.

## 1. Introduction

G protein-coupled receptors (GPCRs) are a large family of membrane proteins that participate in the majority of cell signaling processes in the body. As such, they have been the focus of extensive research efforts over the last few decades [1]. The function of these receptors and the mechanism of activation and downstream signaling are quite well understood [1,2]. Th activation of GPCRs is usually a result of the binding of an extracellular agonist to a specific binding site on the receptor. This binding stabilizes the receptor in its active state, which may activate the G protein-mediated signaling processes in the cells, as well as G protein-independent signaling pathways, e.g., via β arrestin [3].

Membrane potential is known to regulate a wide range of cellular processes. These include the excitability of neuronal cells, as well as cell migration, orientation, and nerve growth. It is also known to affect regeneration, development, and cell proliferation in non-neuronal cells [4]. Membrane potential has known for several decades to regulate membrane proteins. The most studied voltage-sensitive proteins are the voltage gated ion channels. For these proteins, it has been shown that changes in membrane potential cause a conformational change within the channel protein, thereby leading to channel opening and to a flow of ions through the channel pore [5,6]. A weaker voltage dependence was also found in ligand-gated channels such as the N-methyl-D-aspartic acid (NMDA) receptor [7,8], non-NMDA receptors [9] and nicotinic channels [10]. Voltage dependence has been suggested regarding other proteins as well, such as the voltage dependent phosphatase [11], although these possibilities have been studied to a lesser extent. Voltage dependence of signaling processes that are mediated by GPCRs has been demonstrated in several systems, but the notion that GPCRs are intrinsically sensitive to the transmembrane potential mainly become notable only over the past two decades. In this review, we will describe the evidence for the voltage dependence of GPCRs and discuss the possible molecular mechanisms that underlie this phenomenon.

## 2. Voltage Dependence of GPCRs

**Figure 1:**
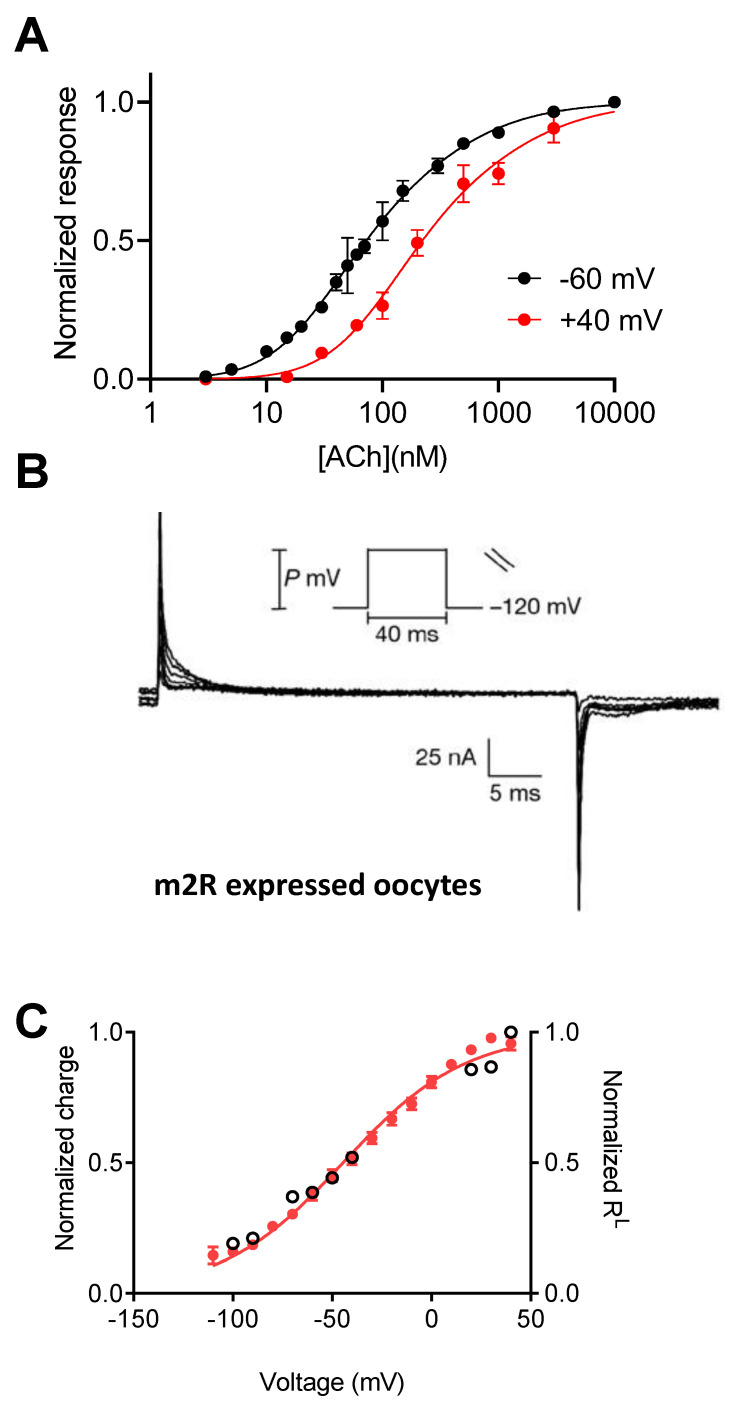
Voltage dependence of the M2R. (**A**) Dose–response curves obtained at -60 mV and +40 mV show a rightward shift under depolarization; taken with permission from [22]. (**B**) Charge movement-associated currents measured from M2R-expressing oocytes; taken from [37]. (**C**) Current-voltage (red) and R^L^-voltage (empty circles) curves (receptors reside in low affinity state) showing a tight correlation between charge movement and shift in affinity; taken from [37].

In the past, several processes mediated by GPCRs were found to be modulated by membrane potential. For example, the release of Ca^2+^ from intracellular stores induced by purinergic receptors was found to be enhanced by membrane depolarization [12,13,14]. Another well-studied example is the voltage-dependent properties of a muscarinic receptor evoked cationic current, activated by carbachol in smooth muscle cells [15,16], and the voltage dependent role of muscarinic receptors in neurotransmitter release [17,18]. These studies were conducted in a physiological setting, where the source of the voltage dependence could not be attributed to the receptor itself. To identify the voltage sensitive molecule, studies were later conducted in more isolated heterologous expression systems. The first GPCR to be investigated for its ability to sense the membrane potential was the M2 muscarinic receptor (M2R). The M2 muscarinic receptor (M2R) has been studied in physiological processes affected by depolarization [18,19,20,21]; therefore, it was a suitable candidate to be investigated for voltage sensitivity. The receptor was expressed in oocytes from *Xenopus laevis*, and M2R-activated GIRK (G protein-activated inward rectifying potassium channel) currents were used as a measure for agonist binding and receptor activation [22]. These measurements revealed that the apparent affinity of acetylcholine (ACh) and oxotremorine toward the M2R was modulated by membrane potential, as it was decreased upon depolarization (Figure 1A). Furthermore, the binding of labeled ACh to M2R-expressing oocytes was lower when the cell was depolarized by elevating extracellular potassium, suggesting that the M2R itself is voltage-sensitive [22]. Similar approaches were further used to show that the M1 muscarinic receptor (M1R) is voltage sensitive as well. Remarkably, the membrane potential affected the binding of ACh to the M1R in an opposite manner; that is, depolarization increased rather than decreased the binding of ACh toward the receptor.

Following this study, similar approaches were taken to study the voltage dependence of other GPCRs. By these means, it was shown that the dopamine D2 receptor [23,24,25], and later on, the D1 and D5 receptors [26], are voltage sensitive as well. For these receptors, it was further shown that the voltage dependence is agonist specific. While the potency of dopamine toward the D2 receptor was voltage dependent, the potencies of the dopaminergic agonists β-phenethylamine, p-tyramine, and m-tyramine were voltage-insensitive [27]. Similar agonist specificity was also later described for other receptors [28,29,30,31]. Interestingly, the same oocyte functional expression system was also used successfully to show voltage dependence in the class C metabotropic glutamate receptors mGluR1 and mGluR3 [32]. The observation that voltage dependence is not confined to class A GPCRs may have mechanistic implications, as will be described below.

Another approach that has been proven efficacious in the study of voltage sensitivity in GPCRs is the direct measurement of GPCR activation using a forester resonance energy transfer (FRET) signal, either between two fluorophores within the GPCR molecule (i.e., between a fluorophore in the c-terminal and a fluorophore in the 3rd intracellular loop of the receptor) or between a fluorophore in the receptor and a fluorophore in an effector molecule (e.g., a G protein subunit or arrestin). Experiments employing this approach confirmed the observations regarding muscarinic receptors and demonstrated voltage dependence in several other GPCRs, including, among others, adrenergic receptors [33], μ-opioid receptors [31], and prostanoid receptors [34].

A step towards demonstrating that GPCRs are intrinsically voltage sensitive came from the measurements of depolarization induced charge movement associated currents. In voltage dependent proteins, such as voltage gated ion channels, changes in the membrane potential result in the movement of charges, leading to conformational change, which, in turn, may affect the function of the protein [35]. These charge movements, manifested as transient capacitive currents, were first described in voltage gated sodium channels, where they were interpreted as movement of the voltage sensor leading to the opening of the channel pore, therefore termed “gating currents” [36]. In GPCR, such currents were first measured in the oocyte, expressing either the M2R or the M1R (Figure 1B), and were similarly termed gating currents, although they do not involve the gating of a channel [37]. The amount of charge that moves upon depolarization was calculated to be less than 1 eV. This low value of gating charge may suggest a weaker voltage dependence (in comparison to ~13 eV in voltage gated channels) [38,39]. Charge–voltage relationship (Q–V curve) extracted from these currents revealed that the charge movement occurs in a physiological range of membrane potentials, with a V_50_ (the membrane potential where 50% of the charge moved) of −44 mV. To check whether the movement of charges underlies the voltage-dependent changes in the affinity of M2 receptors, the Q–V curve was compared to the dependence of the fraction of receptors in a high-affinity state that shifted to a low-affinity state due to membrane potential (R^L^–V curve). Figure 1C shows that the two curves are well correlated, thus suggesting that the two processes are tightly coupled [37].

Following these findings, additional experiments were designed to further investigate the linkage between voltage and conformational changes in the M2R receptor. Dekel et al. [40] simultaneously measured gating currents and voltage dependent conformational changes using voltage clamp fluorometry. By labeling a cysteine in the vicinity of the receptor’s orthosteric binding site, it was found that the receptor undergoes a conformational change near its binding site, and that the voltage dependence of this conformational change correlates well with both the voltage dependence of the gating currents and the voltage dependence of the affinity. Furthermore, treatments that abolished the voltage dependent shift in affinity, such as treatment with pertussis toxin (PTX), abolished the voltage dependent conformational changes, suggesting that these two processes are linked. This cumulative data opened a new line of research focused on elucidating the molecular mechanism that underlies the voltage dependence of GPCRs.

## 3. The Molecular Basis for Voltage Dependence of GPCRs

The mechanism that underlies the voltage-induced activation of the ion channel is understood at the molecular and, to some extent, the structural level [5,41]. However, GPCRs do not contain an obvious voltage sensor that resembles the canonical voltage sensor found in voltage gated ion channels. Therefore, it is likely that a unique molecular mechanism underlies the voltage dependence of these proteins. Several mechanisms were suggested to explain both the observed depolarization induced charge movement (gating currents) and the voltage dependence of the activity and affinity of the GPCRs. Below, we will discuss these putative mechanisms.

## 4. A Tyrosine-Based Voltage Sensor in the M2R

As mentioned above, voltage dependence of a voltage gated ion channel was attributed to the movement of charges that comprise a voltage sensor and are located mainly in the S4 region of the channel. The identification of this voltage sensor was achieved by examining the effect of mutations in some candidate residues on the movement of the gating charges [6,41,42]. Barchad-Avitzur et al. [43] employed a similar approach in an attempt to identify the voltage sensor of the M2R. As the two charged residues in the transmembrane domains of the M2R are either not voltage sensing (D103^3.32^) [29], or not responsible for the voltage dependence of agonist binding (D69^2.50^, [44]), the authors examined the possibility that the voltage sensor could be comprised of polar residues. In particular, tyrosine has a strong intrinsic dipole moment [45]; therefore, it was proposed that it may sense changes in membrane potential by reorienting its polar side chain [41].

**Figure 2:**
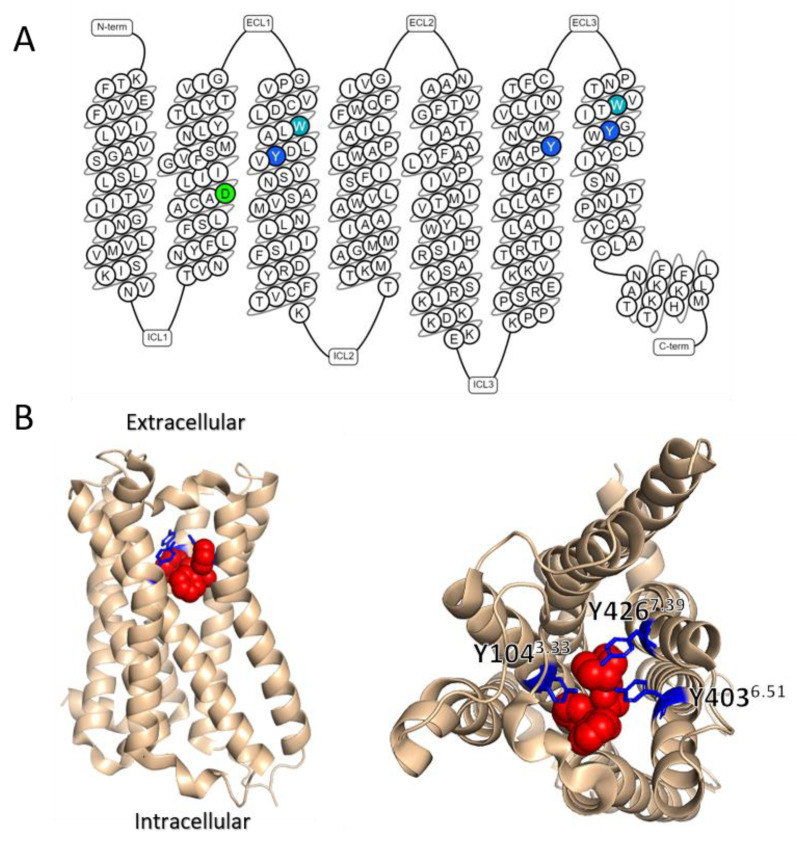
(**A**) Snake-like plot of the M2R (generated by GPCRdb.org [1]). Residues implicated in voltage sensing are labeled as follows: D69^2.50^ (green), residues of the tyrosine lid (blue), and residues of the allosteric site (cyan). (**B**) Side (left) and top (right) view of the M2R based on structure PDB ID: 3OUN [50]. Here and in Figure 3, the antagonist QNB is shown in red to indicate the ligand binding site. The three tyrosine residues that form the tyrosine lid are labeled in blue.

The study of Barchad-Avitzur et al. focused on three tyrosine residues (Y104^3.33^, Y403^6.51^, Y426^7.39^) that are located in the transmembrane domains of the M2R and other muscarinic receptors (Figure 2A,B). Their study found that these three tyrosine residues indeed play a role in the voltage dependence of the M2R. Specifically, mutating each one of these tyrosine residues to either alanine or phenylalanine resulted in a decrease in the measured charge moving per receptor. Furthermore, these mutants exhibited a reduced voltage-induced conformational change and diminished the voltage dependence of M2R- induced GIRK currents. These results suggested that the three tyrosines form a voltage-sensing element in the M2R, and perhaps in the M1R as well. Interestingly, mutating these three tyrosine residues in the *Drosophila melanogaster* muscarinic type A receptor (which is homologous to M1R in mammalians and exhibits similar voltage dependence) showed low affinity and voltage independence in a similar manner [46].

Importantly, these three tyrosine residues are located in close proximity to the orthosteric binding site of the M2R, and they form a lid that surrounds the amine of the orthosteric ligand [47]. This lid has been suggested to play a crucial role in controlling both agonist binding and G protein coupling [47,48].

Based on these findings, a mechanism by which the three voltage sensing tyrosines control the voltage sensitivity of agonist binding to the M2R was proposed. It was hypothesized that at resting potential, the tyrosine lid is maintained in a closed conformation. When in this state, the receptor exhibits high agonist binding affinity. When depolarization induces movement of the tyrosine residues by way of their hydroxyl group, the lid adopts an open conformation and consequently, the receptor shifts into a low affinity state. Since the conformation of the tyrosine lid determines both the agonist binding affinity and the extent of G protein coupling [47], the tyrosine-based voltage sensor might be accountable for both the effect of G protein coupling on the voltage dependence of agonist binding affinity and the effect of voltage on the receptor-G protein coupling [48] (see below).

The above mechanism, proposed for the muscarinic receptors, may not be general to other GPCRs, as a tyrosine lid is present only in muscarinic receptors. However, it is possible that voltage-sensing residues in the vicinity of the binding site serve a similar role in other receptors. For example, it has been suggested that activation of the β-adrenergic receptor, which also exhibits voltage dependence [49], is accompanied by the closure of a lid above the ligand binding site of the receptor [48]. The role of this lid in the voltage dependence of these receptor is yet to be determined.

## 5. Role of the Ligand Binding Site in Determining the Voltage Dependence of GPCRs

The observation that voltage induces conformational change in the agonist binding site, and that in many cases the voltage dependence of GPCR is ligand-dependent, led to the suggestion that the binding site itself may participate in voltage sensing. Navarro-Polanco et al. [29] analyzed the effect of mutations in several residues implicated in ligand binding (D103^3.32^, W99^3.28^, and Y104^3.33^ in TM3, and Y403^6.51^ in TM6) on the gating currents. While these mutations did not affect the slope of the Q–V relationship (which may reflect the amount of gating charge; see [51]), they did shift the V1/2 of the Q–V curve. These authors concluded that these residues allosterically influence the voltage sensor movement rather, than function as the primary voltage sensor itself [29]. Rinne et al. further analyzed the voltage sensitivity of the M1, M3, and M5 muscarinic receptors in order to find structural correlates for the differential effect of voltage on different agonists [30]. In an elegant series of FRET-based experiments, combined with molecular docking analysis, they found that the effect of voltage on activating the receptors is determined by the mode of agonist binding to the receptor. They demonstrated that different ligands adopt different binding poses when interacting with the binding pocket, and this binding mode may influence the way that voltage affects the activation of the receptor by a given agonist. They further showed that a mutation of a single residue (N514^6.58^) affected the voltage dependence of the activation of one ligand, but not of other. These findings provide structural insight into the effect of voltage on the conformation of the ligand binding site. The way by which voltage induces such an effect remains to be studied.

## 6. G Protein Coupling as a Mechanism for Voltage Dependence of GPCRs

Some studies have hypothesized that G protein coupling is also involved in the voltage dependence of GPCRs, in particular, the muscarinic receptors. This suggestion is based on experiments showing that following treatment of the M2R with pertussis toxin (which uncouples the receptor from its G protein), its binding affinity was low and the voltage dependence of the binding of ACh was abolished [22]. Furthermore, switching the entire 3rd intracellular loop (L_3_, a region known to take part in G protein-coupling) between the M2R and M1R reversed the direction of the voltage sensitivity of the receptor (i.e., M2R with the L_3_ of the M1R showed voltage sensitivity similar to that of the WT M1R, and vice versa) [37]. Finally, mutation of five residues of this region abolished both the voltage dependency of conformational change and the voltage dependency of agonist binding to the M2R [37,40].

Notably, this mutation did not affect the charge movement of this receptor, leading to the conclusion that these residues, while involved in determining the voltage dependence of the agonist binding and receptor activity, are not part of the voltage sensing mechanism in the M2R.

More recently, we have shown that the G protein-coupling itself is affected by voltage, even in the absence of agonist binding [52]. This was demonstrated by evaluating the constitutive activity of the M2R from agonist-independent M2R-activated GIRK currents. It was found that this activity is voltage dependent; it is higher at resting potential than under depolarization, thus leading to the conclusion that the coupling of the M2R to its G protein is, by itself, voltage dependent. The involvement of G protein coupling in the voltage dependence of GPCRs has also been examined for the α2 adrenergic receptor [33]. In this case, pretreatment with PTX did not alter the voltage dependence of the intracellular FRET signal induced by the application of the agonist. The authors of this study concluded that the voltage dependence of the α2 adrenergic receptor does not depend on G protein-coupling. It is worth noting that in this experimental design, the FRET probes are introduced in regions of the receptor that may be involved in G protein-coupling, and therefore, it is possible that the behavior of this construct may not fully reflect the behavior of the native receptor.

## 7. The Involvement of Sodium Ions in the Voltage Dependence of GPCRs

**Figure 3:**
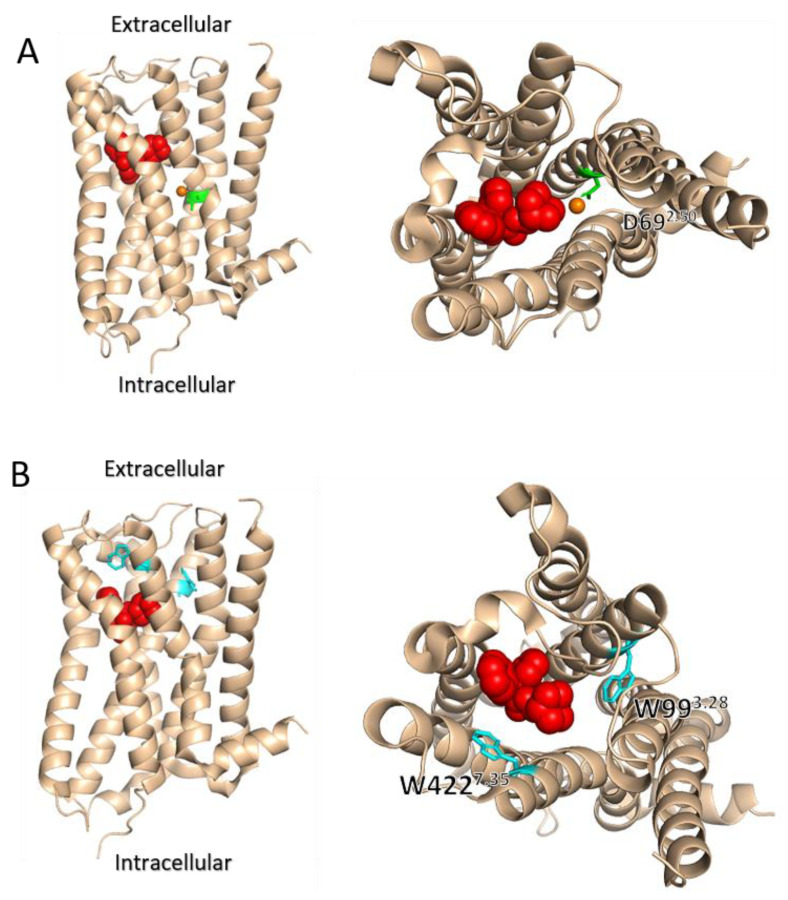
Side (left) and top (right) view of the M2R bound to an antagonist (red). In (**A**) Na^+^ ion is shown in orange in its predicted location, and residue D69^2.50^ (which was implicated in Na^+^ binding) is shown in green. In (**B**), two residues that are part of the allosteric site and were implicated in voltage sensing are shown in cyan.

It is well established that Na^+^ has an allosteric effect on GPCRs. Several studies have shown that Na^+^ modulates the affinity and activity of many GPCRs, including dopaminergic, adrenergic, and adenosine receptors [53,54,55] (reviewed in [56]). Recent structural studies in several GPCRs, including the A2a adenosine receptor, the β1 adrenergic receptor, and the δ opioid receptor [57,58,59], have found an Na^+^ ion in a defined binding site located in the helical bundle, with a conserved aspartate in position 2.50 (Ballesteros and Weinstein numbering [60]) playing a role in Na^+^ binding (Figure 2A and 3A). These studies confirmed earlier findings in which this residue was found to play a role in the allosteric modulation of GPCRs by Na^+^ [61,62,63,64]. Recently, theoretical simulations suggested a link between the binding of Na^+^ and the voltage dependence of GPCRs [65,66]. According to these studies, the depolarization of the cell membrane induces movement of an Na^+^ ion (or other cations) that is bound to a binding site within the transmembrane domain towards the extracellular side. The experimentally measured gating charge that moves in the M2R during depolarization is in agreement with the value obtained in the theoretical simulation for this transition. Upon repolarization, the Na^+^ ion moves back into its binding site. The results of these simulations are consistent with the observation that mutating D^2.50^ in the M2R (D69^2.50^) diminishes the gating current. Note, however, that the charge movement-associated currents were measured experimentally in the absence of Na^+^ in both the intracellular and extracellular solutions (Na^+^ was replaced by the large ion N-Methyl-D-glucamine (NMDG)), thus making the above described scenario somewhat less likely, while it is possible that the observed charge movement is due to the movement of this large cation. While voltage-induced cation movement may provide a robust and general mechanism for the voltage dependence of family A GPCRs, some experimental evidence is not consistent with such a mechanism. First, a recent study has shown that replacement of Na^+^ with NMDG in the extracellular solution affects the potencies of ACh toward the M2R and of serotonin toward the 5-HT1A receptor, but does not affect their voltage-dependence [67,68]. Second, the neutralizing of D69^2.50^ in the M2R receptor did not abolish either the voltage induced conformational change in the M2R [43] or the voltage dependence of the receptors potency in the M2R [44]. Similar results were also obtained by mutating D82^2.50^ at the 5-HT1A receptor [68]. Furthermore, such a mechanism is unlikely to account for the voltage dependence of the family C mGluRs, in which voltage dependence has been reported [32].

## 8. The Role of the Allosteric Binding Site in the Voltage Dependence of GPCRs

The observation that depolarization may exert opposite effects on different receptors and on the potency of different ligands for the same receptor led to the notion that membrane potential may be viewed as an allosteric modulator of GPCRs. Under this framework, Hoppe et al. [69] compared the effect of voltage to the effect on allosteric modulators. Using FRET measurements from the M1R and M3R, they examined the effects of alterations in the allosteric binding site of these receptors, either by the exchange of different regions between the receptors, or by point directed mutagenesis. They found that the allosteric binding site plays a role in determining the voltage dependence of these receptors. The experimental evidence was also corroborated by molecular dynamic simulations that suggested a possible role for the interaction between residues in the allosteric binding site to residues in the orthosteric binding site, such as the aforementioned tyrosine lid. This notion is also compatible with previous results obtained for the M2R. In this receptor, it was found that mutating two residues that are located in the allosteric binding site, W422^7.35^ and W99^3.28^ (Figure 2A and Figure 3B), abolished the voltage dependence of agonist affinity [40]. Interestingly, these mutations did not significantly affect either the depolarization-induced conformational change or the depolarization induced charge movements. These results suggest that the allosteric site does not play a role in voltage sensing or in the conformational change accompanying it; rather it plays a role in determining the potency of the agonist in activating the receptor. Together, these result illustrate a complex interplay between the voltage sensor, the orthosteric binding site, and the allosteric binding site in determining the extent and direction of the voltage dependence.

## 9. A Thermodynamics Perspective on Voltage Dependence of GPCRs

While most studies explore the molecular nature of the voltage dependence of GPCRs, it is possible to view the GPCR and the membrane where it resides in thermodynamic terms. From such a point of view, we may assume that a GPCR can reside in multiple ground states and active states. The distribution between these states is dictated by the free-energy term that is associated with the transition between these states. The binding of a ligand will enhance, by definition, the transition to the active states. The membrane potential can serve as an energy source of the cell and as such, may affect the conformation of the GPCR and the free energy associated with ligand binding and receptor activation, This is consistent with the agonist specificity of the voltage dependencies of ligands [27,29,30,31], since voltage may induce a conformational change that will energetically favor the binding of a given ligand, but not others.

A proposed model considered the process of voltage induced conformational change as one that requires a source of charges and a path on which the charge can move. Accordingly, it was hypothesized that the voltage sensor in GPCRs may not be a defined region within the receptor, nor a specific residue(s). Rather, the voltage dependence of a GPCR may be a result of the overall charge distribution of the protein. A network of conserved hydrogen bonds in the transmembrane domain was suggested to exist in the GPCRs [70,71,72,73,74], and this may serve as the path for the charge. It is interesting to note that such a network is not common in other membrane proteins [75]. Thus, it has been postulated that this conserved network may play a role in charge transfer during GPCR activation [76]. In addition, some residues within the network are able to change their charge status. Thus, this hydrogen bond network may potentially provide a path for charge movement (e.g., a Grotthuss proton wire [77]) within the transmembrane core of the GPCR. In addition, the location of this network in the vicinity to the ligand binding sites is also consistent with its postulated role in affecting both ligand binding and activation. Currently there is no experimental data to support the role of such a network in voltage dependence. The exploration of such a role, for example, of the two conserved motifs (the CWxP motif and the NPxxY motif [71]) that were proposed to be part of that network, may shed new light on such a possible mechanism.

## 10. Physiological Roles of Voltage Dependence of GPCRs

Given the role of GPCRs in almost any physiological function and their abundant expression in excitable cells, it is likely that the voltage dependence of GPCRs has physiological significance. The observation that charge movement occurs in a physiologically relevant membrane potential range and the fast kinetics of these charge movements suggests that even a single action potential may alter the function of a GPCR. The dual function of GPCRs as both ligand and voltage sensors presents a novel and unique mechanism of synergistic effects of electrical and chemical signals. Moreover, owing to this characteristic, it is conceivable that these receptors may serve as coincidence detectors, thus participating in processes that are related to learning and memory. Several fundamental cellular processes have been investigated in this context. A well-studied example is the process of neurotransmitter release. As mentioned above, the study of voltage dependence of the M2R originated from the hypothesis that an additional voltage dependent process, besides the opening of a voltage gated calcium channel, controls the neurotransmitter release from fast synapses. According to that hypothesis [17,78,79], at resting membrane potential, presynaptic auto-receptors, such as the M2R and the metabotropic glutamate type 3 (mGlu3) receptors, interact with the protein of the release machinery and thereby inhibit transmitter release. Following the arrival of the action potential, the presynaptic terminal is depolarized. This change in membrane potential leads to a conformational change in the receptor that will decrease the affinity of the receptor to the neurotransmitter, weaken the interaction of the receptor with the release machinery, and thus evoke transmitter release. Some experimental evidence support such a scenario. It has been shown in brain synaptosomes that the M2R interacts with proteins of the release machinery, and that this interaction is voltage-dependent; it is weaker under depolarization [80]. Furthermore, it was shown in the same experiment that the binding of ACh to the M2R is weaker under depolarization [81]. A more direct evidence to support this scenario has recently been obtained [82]. In this study, the abolishment of depolarization induced charge movement in the M2R affected the amount and timing of transmitter release. These results suggest that the direct effect of voltage on the M2R plays a role in controlling this fundamental neuronal process.

The physiological role of voltage sensitivity of the M2R has also been demonstrated in cardiac cells. In these cells, M2R-mediated cardiac GIRK currents (I_KACh_) exhibit a property known as “relaxation”, which refers to a slow decrease or increase in current magnitude with depolarization or hyperpolarization, respectively. This relaxation depends on agonist concentration, as it is observed only at low agonist concentrations [83]. Recent studies suggest that the voltage dependence of agonist binding to the M2R underlies the relaxation of cardiac GIRK currents. According to this suggestion, hyperpolarization increases the affinity of ACh toward the M2R, thereby contributing to increased I_KACh_ at hyperpolarized membrane potentials. Conversely, depolarization decreases the affinity of the receptor, thus decreasing I_KACh_ [83,84].

While the above examples demonstrate the physiological role of GPCRs voltage dependence in various cellular processes, a recent study verified that the voltage dependence of GPCRs contributes to neuronal coding and behavioral output under physiological conditions in vivo as well [46]. This study used *Drosophila* as a model to show that muscarinic mediated neuronal potentiation in vivo is voltage dependent and that this voltage dependent potentiation is abolished in mutant animals expressing a voltage independent type A muscarinic receptor. Furthermore, this muscarinic receptor voltage independent mutant showed a strong behavioral effect of increased odor habituation.

Voltage dependence of GPCRs may provide an explanation for previously observed behaviors that involve GPCRs. For example, we have recently reported voltage dependence of the cannabinoid 1 (CB1) receptor [85]. We may hypothesize that the observed agonist specificity of the voltage dependence of this receptor may underlie the different roles 2-AG and AEA play in some forms of synaptic plasticity. Specifically, it was suggested that AEA is less effective than 2-AG in inducing depolarization induced suppression of excitation (DSE) [86,87]. Our observation that the potency of 2-AG is higher under depolarization may suggest that under depolarization, a low 2-AG concentration is sufficient in order to activate the CB1 receptor and thereby inhibit release. The potency of the second endocannabinoid, AEA, was shown to be voltage independent. Thus, the potency of AEA is expected to remain low, even under depolarization, and therefore. AEA would be less effective in inducing DSE.

To conclude, while voltage dependence has been shown for many GPCRs, the molecular mechanism that underlies it is not yet well understood. This review described the evidence supporting some possible underlying molecular mechanisms. Further studies are needed in order to achieve a more comprehensive view of how these different mechanisms act together to obtain GPCRs with their voltage dependence, as well as to elucidate the physiological roles of this voltage dependence.
